# Anti-Stress, Glial- and Neuro-Differentiation Potential of Resveratrol: Characterization by Cellular, Biochemical and Imaging Assays

**DOI:** 10.3390/nu12030671

**Published:** 2020-02-29

**Authors:** Sajal Afzal, Sukant Garg, Divya Adiga, Yoshiyuki Ishida, Keiji Terao, Sunil C. Kaul, Renu Wadhwa

**Affiliations:** 1 DAILAB, DBT-AIST International Center for Translational and Environmental Research (DAICENTER), National Institute of Advanced Industrial Science & Technology (AIST), Tsukuba 305-8565, Japan; sajal.afzal@aist.go.jp (S.A.); sukantgarg@gmail.com (S.G.); divyaadiga1993@gmail.com (D.A.); 2 School of Integrative and Global Majors, University of Tsukuba, Tsukuba 305-8577, Japan; 3 Manipal School of Life Sciences, Manipal Academy of Higher Education (MAHE), Manipal 576-104, India; 4 CycloChem Co., Ltd., 7-4-5 Minatojima-minamimachi, Chuo-ku, Kobe 650-0047, Japan; yoshiyuki.ishida@cyclochem.com (Y.I.); keiji.terao@cyclochem.com (K.T.)

**Keywords:** Oxidative stress, DNA damage, hypoxia, protein aggregation, old age, resveratrol, differentiation, protection

## Abstract

Environmental stress, exhaustive industrialization and the use of chemicals in our daily lives contribute to increasing incidence of cancer and other pathologies. Although the cancer treatment has revolutionized in last 2–3 decades, shortcomings such as (i) extremely high cost of treatment, (ii) poor availability of drugs, (iii) severe side effects and (iv) emergence of drug resistance have prioritized the need of developing alternate natural, economic and welfare (NEW) therapeutics reagents. Identification and characterization of such anti-stress NEW drugs that not only limit the growth of cancer cells but also reprogram them to perform their specific functions are highly desired. We recruited rat glioma- and human neuroblastoma-based assays to explore such activities of resveratrol, a naturally occurring stilbenoid. We demonstrate that nontoxic doses of resveratrol protect cells against a variety of stresses that are largely involved in age-related brain pathologies. These included oxidative, DNA damage, metal toxicity, heat, hypoxia, and protein aggregation stresses. Furthermore, it caused differentiation of cells to functional astrocytes and neurons as characterized by the upregulation of their specific protein markers. These findings endorse multiple bioactivities of resveratrol and encourage them to be tested for their benefits in animal models and humans.

## 1. Introduction

Cultured human cells show stress response to a variety of environmental stimuli including chemical toxins, temperature variations, radiations and changes in oxygen and pressure. Based on the experimental evidence that these molecular responses are similar to those that occur at the tissue and organismic level, cell culture-based discovery of anti-stress reagents has been promoted in food, drug and cosmetic industries [1]. A variety of stress models have been in use to (i) investigate stress-induced biomolecular signaling, and (ii) discover reagents to intervene these changes and thus develop anti-stress reagents. Oxidative stress in cellular niche results from the accumulation of abnormally generated reactive oxygen species. It disrupts redox signaling and causes DNA double-strand breaks, mitochondrial dysfunction and deleterious effects in the downstream protein signaling [2]. Several cell culture-based models have been developed to generate and mimic the effects of a variety of stresses, such as benzopyrene (cigarette smoke) [3], cobalt chloride (hypoxic stress) [4], variations in temperature (heat stress) and arsenite (heavy metal) [5], adrenaline or epinephrine (shock-response) [6]. Most of these stresses have been shown to induce accelerated biological ageing and diseases such as cancer and neurodegeneration [7]. There is an increase in the incidence of these diseases because of greater use of chemicals and exposure to environmental stressors; leading global health agencies have predicted their prevalence to be alarming in the near future [8].

Chronic stress has been shown to cause a variety of diseases of which cancer, the disease of proliferation, is the most complex and lacks effective therapeutic modalities due to limitations in surgical, radiotherapy procedures and blood-brain barrier during the chemotherapy [9,10]. In the present study, we have recruited glioma derived from astrocytic cancer, possessing the capability of differentiation into the astrocytes with distinct star-shaped morphology and expression of specific hallmark proteins (GFAP and Nestin) [11]. They play a central role in the regulation of cell anchorage, nutrition and repair [12]. Collectively, the gliomas comprise 30% of all brain cancers and 80% of all the brain malignancies with a very poor prognosis and an unclear cause that warrant urgent attention [13]. Since glioma and other closely-related types of cancer progress in a large window of time, these are often termed as diseases of the old age. Timely interventions to prevent stress and toxicity may prove to be useful in preventing or delaying the disease. On the other hand, reprogramming of cancer cells to functional differentiated cells may serve as a mild and effective therapy [5,14,15]. Reprograming glial cells by manipulating their biochemical properties has been suggested to flourish into valuable therapeutic opportunities [16]. Some recent studies have reported the differentiation of glioma cells into neurons [17,18]. Various targeted synthetic anticancer compounds have been introduced in the last few decades, however, a majority of them suffer drawbacks concerning severe toxic effects, commercial cost, and appearance of drug-resistant forms [19]. Natural compounds, as appreciated since ancient times, are gaining importance because of (1) higher tolerability, (2) wider spectrum of the mechanism of action, (3) lower cost and (4) easy and abundant availability [14,20]. Abiding consumption of some natural compounds has been claimed to control the burden of various long-term illnesses [21,22]. A number of natural compounds have been shown to act as anti-stress agents to counter the effects of oxidative stress [10] urging further studies to validate them as NEW (natural, economic and welfare) preventive and therapeutic drugs. 

Resveratrol (trans-3,4,5’-trihydroxy-stilbene) is a naturally occurring low molecular weight (228.25 g/mol) polyphenolic phytoalexin, commonly produced and found in a variety of foods in response to exposure to various environmental stimuli [23,24]. It is known to possess hepato-protective, anticancer, and cardio-and neuro-protective potential [25,26] that have been attributed to its potent antioxidant properties [27]. In various in vitro and in vivo models, resveratrol has been shown to protect against a number of stresses [28,29,30,31,32,33]. It was shown to induce growth arrest and apoptosis in cancer cells at high doses (> 10 µM) [34] and also cause inhibition of cancer cell metastasis by multiple pathways involving mitochondrial dysfunction, ROS (reactive oxygen species) generation and activation of DNA damage and Hedgehog signaling [29,30,31]. Wang et al. showed inhibition of hypoxia-induced cell migration and invasion in multiple glioma cell lines. It was mediated by the suppression of STAT3 phosphorylation at 20–40 μM dose in a time-dependent manner [33]. However, Gweon et al. reported an increase in the migration potential of the HT1080 fibrosarcoma at doses up to 50 μM by upregulation of MMP9 and suppression of MAPK/PI3K pathways [35]. Resveratrol was shown to induce cell growth arrest and promote glial- and neuronal-type differentiated morphology at 100 μM dose in human glioblastoma U87MG cells [28]. These contradictory expositions suggest dose-dependent or cell-type-specific activities of resveratrol and have indeed posed difficulties to categorize it as an anti-apoptotic, pro-apoptotic, anti-migratory or pro-migratory molecule. For old age-related diseases, resveratrol was shown to ameliorate symptoms of Alzheimer’s disease and dementia via inhibition of the oligomeric amyloid β-induced expression levels of phosphorylated p47 and phosphorylated gp91 and increased hippocampal neurogenesis and angiogenesis [36,37]. Owing to the contribution of a variety of stresses such as radiations, heavy metal and heat in old age pathologies such as amyloidosis, tauopathies, synucleinopathies and TDP-43 proteinopathies, we first determined the dose of resveratrol that could protect cultured glial cells against a large range of stresses. Furthermore, we report that this low dose of resveratrol caused functional differentiation of glioma to astrocytes and neurons as defined by expression of specific protein markers. 

## 2. Materials and Methods

### 2.1. Cell Lines and Reagents

Rat Glioma C6, human neuroblastoma TGW, GOTO and IMR32, and normal lung fibroblasts TIG3 cells, were obtained from Cell Resource Center for Biomedical Research, Tohoku University, Sendai, Japan, and Japanese Collection of Research Bioresources Cell Bank (JCRB), Japan. Cells were cultured in Dulbecco’s modified Eagle’s medium (Invitrogen)—supplemented with 5% fetal bovine serum and 1% penicillin/streptomycin in a humidified incubator (37 °C and 5% CO_2_). Resveratrol was purchased from Tokyo Chemical Industry Co., Ltd., Japan (R0071), dissolved in DMSO to make 100 mM stock and stored at −20 °C. Hydrogen peroxide, cobalt chloride, epinephrine, benzopyrene, and sodium (meta)arsenite were dissolved in phosphate-buffered saline (PBS) and used for various assays as indicated. A UV chamber (FS-800, FUNA^^®^^-UV-linker) was used to expose the cells to UV radiations (UV-C (254 nm) at 100 V and 8W energy). UV energy was monitored in tests of mJ/cm^2^ (1 mJ/cm^2^ = 1 mW. second/cm^2^). A humidified CO_2_ incubator (maintained at 42 °C and 5% CO_2_) was used to induce heat stress. Primary antibodies against Luciferase (AbCam, ab16466), γH2AX (Cell Signaling, 9718S), pCHK1 (Cell Signaling, 2344S), pCHK2 (Cell Signaling, 2197P), HP1γ (Merck Millipore, 05–690), Mortalin [38], β-actin (AbCam, ab49900), GFAP (Sigma-Aldrich, G9269), Nestin (Santa Cruz, SC-23927), MAP2 (Sigma-Aldrich, M3696), PSD-95 (Santa Cruz, SC-32290), Neuropsin (Santa Cruz, SC-134600), GAP43 (Santa Cruz, SC-33705), NCAM (Santa Cruz, SC-10735), Survivin (Santa Cruz, SC-10811), Vimentin (Santa Cruz, SC-6260), NeuN (AbCam, ab177487), TCF4 (Santa Cruz, SC-166699), SMAD2/3 (Cell Signaling, 8685S), pSMAD2/3 (Cell Signaling, 8828), hnRNP-K (Cell Signaling, 4675S), E-cadherin (Cell Signaling, 5296S), and MMP2 (Santa Cruz, SC-13594) proteins were used for immunostaining or blotting as indicated.

### 2.2. Cytotoxicity Assays

Cells (2000/well) were plated in 96-well plates, allowed to settle overnight, and then treated with varying doses of resveratrol/stressors. The control (DMSO) or treated cells were incubated for 48 h followed by addition of 10 µL of PBS containing 5 mg/mL MTT (3-(4,5-dimethylthiazol-2-yl)-2,5-diphenyltetrazolium bromide) (Life Technologies, M6494), and further incubated for 4 h. Culture medium containing MTT was replaced with DMSO and mixed thoroughly, followed by measurement of optical density at 570 nm using Tecan infinite M200^^®^^ Pro microplate reader (Tecan Group Ltd., Mannedorf, Switzerland). Cell viability was calculated as a percentage against the control, and various IC values (IC_10_, _20_ and _50_- the doses that killed 10, 20 and 50% of the cells) were determined using Microsoft™ Office^© 2020^.

For visual analysis, cells (5000 cells/well) were plated in 6-well plates and allowed to settle overnight, followed by culture either in control or resveratrol-supplemented medium. Cells were fixed in each variant of treatment, stained with 0.5% crystal violet, washed and air-dried at room temperature for 48 h followed by de-staining using 15% methanol and acetic acid solution for 1 min, and quantification by absorbance measurement at 570 nm. A proliferation histogram was plotted considering control on day 1 as 100 per cent.

### 2.3. Anti-Stress Activity Assay

Cells (2000/well) were plated in two 96-well plates and allowed to settle overnight. Various stress conditions were applied as indicated followed by cytotoxicity assay as described above. Cells were then subjected to stress conditions yielding 20% cytotoxicity and recovered in either control or resveratrol (2 μM)-supplemented medium for 48 h. Viability was determined by MTT assay as described above. Cell viability in control was taken as 100%. The experiment was performed three times, and the histograms bearing cumulated data were plotted.

### 2.4. ROS Assay

Cells (25 × 10^3^/well) were plated in 12-well plates, allowed to settle overnight, and treated with 2 μM resveratrol. After 24 h of pretreatment, the cells were stressed with 200 μM H_2_O_2_ for 2 h and recovered either in control or resveratrol (2 μM)-supplemented medium. 500 μM H_2_O_2_ was taken as a positive control. The extent of ROS induction in cells was evaluated using DCFDA stain in Image-IT^TM^ LIVE Green Reactive Oxygen Species Detection Kit following the manufacturer’s recommendations. ROS (labelled in green) intensity was calculated by ImageJ software (NIH, 1.52a) and tabulated as a percentage against the control using Microsoft™ Office^© 2020^. ROS intensity in stressed cells (200 μM H_2_O_2_) was taken as 100%.

### 2.5. Comet Assay

Cells (10 × 10^4^/well) were plated in 6-well plates, allowed to settle overnight, and treated with 2 μM resveratrol. After 24 h of pretreatment, the cells were stressed with UV radiation at the rate of 2 mJ/cm^2^ and recovered in 2 μM resveratrol-supplemented medium. 10 mJ/cm^2^ UV was taken as a positive control. The extent of UV-mediated DNA damage was evaluated using a single cell gel neutral comet assay (Trevigen’s Comet Assay^^®^^, 4250–050-K) electrophoresis system following the manufacturer’s protocol. Comet tail length was calculated by ImageJ software (NIH, 1.52a) and tabulated as a percentage against the control using Microsoft™ Office^© 2020^. DNA tail length in stressed cells (2 mJ/cm^2^ UV) was taken as 100%.

### 2.6. Luciferase Assay

Cells (10 × 10^4^/well) were plated in 6-well plates in two identical sets and allowed to settle overnight. The cells were transfected with pGL4-Luc plasmid expressing a full-length Luciferase driven by a constitutive promoter for 48 h, as described previously [5]. Cells were treated with/without 2 μM resveratrol for 4 h, followed by either heat-stress at 42 °C and 5% CO_2_ or treatment with 200 μM cobalt chloride (hypoxia) for 2 h, and recovery at 37 °C either in the control or 2 μM resveratrol-supplemented medium for another 24 h. The first set of cells were taken for immunocytochemistry for Luciferase protein as described later and the second was lysed using passive lysis buffer for Luciferase expression estimation using the Luciferase Assay System (Promega, E1501) following the manufacturer’s protocol. The expression of the encoded luciferase protein was read as units of luminescence generated in the enzymatic reaction using luciferin as a substrate. Protein expression was quantified by ImageJ software (NIH, 1.52a) and tabulated with the reporter Luciferase luminescence values as a percentage against the control using Microsoft™ Office^© 2020^. Luciferase activity (luminescence) in control was taken as 100%.

### 2.7. Catalase Assay

Cells (2 × 10^5^/well) were plated in 6-well plates and allowed to settle overnight followed by culture of cells in medium with/without 2 μM resveratrol (pretreatment) for 24 h. The next day, the cells were exposed to 30 μM benzopyrene or 60 µM epinephrine for 2 h, followed by recovery in control or 2 μM resveratrol-supplemented medium for 48 h. An amount of 250 μM benzopyrene and 300 μM epinephrine were taken as positive controls. Cells were scraped using a cell scraper, resuspended in 200 μL cold PBS-supplemented with 1 mM EDTA, lysed by sonication for 20 min, and centrifuged at 10000 rpm at 4 °C for 15 min. Formaldehyde concentration in μM in cell lysates was determined using the Catalase Assay Kit (Cayman Chemical, 707002) following the manufacturer’s protocol, and the catalase activity was calculated in nmol/min/ml and plotted against the control using Microsoft™ Office^© 2020^. 

### 2.8. Protein Aggregation and De-Aggregation Assay

Cells stably expressing GFP protein were prepared as previously described [5]. The 5 × 10^4^ cells/well were plated in 6-well plates, allowed to settle overnight, and pretreated with 2 μM resveratrol. After 24 h, the cells were stressed with sodium (meta)arsenite 20 μM for 24 h, followed by three thorough washes with PBS. After washing, cells were recovered in the drug-supplemented medium for 48 h. An amount of 150 μM sodium (meta)arsenite was taken as a positive control. Cells were then visualized under a fluorescent microscope and recorded at 400× magnification. Aggregates were quantified using ImageJ software (NIH, 1.52a) and plotted as a percentage using Microsoft™ Office^© 2020^. The total size of aggregates in stressed cells (20 μM sodium (meta)arsenite) was taken as 100%.

### 2.9. Cell Differentiation

Cells (2000/well) were plated in 6-well plates, allowed to settle overnight, and treated with varying doses of resveratrol/stressors. The resveratrol-supplemented medium was replaced every alternate day for 4–10 weeks. Cells were then visualized under a phase-contrast microscope, recorded at ×400 magnification and taken forward for protein analyses. The number of cells showing differentiated morphology out of 100 cells from 3 random fields were counted manually and tabulated and averaged using Microsoft™ Office^© 2020^. Histograms were plotted against the number of cells with morphological similarity in control taken as 100%.

### 2.10. Western Blotting

Cells (2 × 10^5^/well) were plated in 6-well plates and allowed to settle overnight, followed by the treatment with varying doses of resveratrol/stressors. Control and treated cells were harvested and washed with PBS (× 2), followed by lysis in RIPA buffer (Thermo Fisher Scientific, 89900) containing complete protease inhibitor cocktail (Roche, 4693159001) on ice for 45 min. Lysates were separated on an SDS-polyacrylamide gel using Mini-Protean^^®^^ Tetra cell equipment (Bio-Rad, Hercules, CA, USA), and subjected to western blotting using protein-specific antibodies as indicated and horseradish peroxidase-conjugated secondary HRP antibody (Thermo Fisher Scientific, 31430, 31402 or 31460). Blots were developed using chemiluminescence solution (GE Healthcare, Buckinghamshire, UK) and visualized using a Lumino Image Analyzer (LAS 3000-mini; Fuji Film, Tokyo, Japan). Band intensity was quantified using ImageJ software (NIH, 1.52a) and plotted as a percentage using Microsoft™ Office^© 2020^. Relative band intensity in control was taken as 100%.

### 2.11. Immunostaining

Cells (25 × 10^3^/well) were plated on glass coverslips placed in 12-well cell culture plates. After 48 h of treatment with resveratrol/stressors, control or treated cells were fixed in methanol: acetone (1:1). Cells were permeabilized with Tween-20 in PBS (PBST), washed with PBS, and blocked with 2% bovine serum albumin protein dissolved in PBST. Fixed cells were incubated with primary antibodies (as indicated) overnight, washed with PBS-PBST-PBS (5 min each), incubated with either Alexa-Fluor 488 goat anti-mouse IgG (Life Technologies, A11029), Alexa-Fluor 594 rabbit anti-goat IgG (Life Technologies, A11078) or Alexa-Fluor 594 goat anti-rabbit IgG (Life Technologies, A11037), depending upon the source of the primary antibodies, for 2 h, washed with PBS-PBST-PBS (5 min each), incubated with Hoechst 33342 stain (Invitrogen^^®^^, H3570) for 10 min, washed with PBST-PBS-ultrapure water (5 min each), and mounted on glass slides. The cells were then visualized for immunofluorescence under a microscope at 400 X magnification. Protein expression was quantified using ImageJ software (NIH, 1.52a) and plotted as a percentage using Microsoft™ Office^© 2020^. Relative fluorescence in control was taken as 100%.

### 2.12. Wound Scratch Migration Assay

Cells (25 × 10^4^/well) were plated in 6-well plates and allowed to settle overnight. Cells were then scratched uniformly with the help of a pipette tip, washed thoroughly, and treated with either 2, 8 or 32 μM resveratrol-supplemented medium for 72 h. Cell pictures were taken every 24 h by a phase-contrast microscope. The girth of the gap (scratch) was calculated using ImageJ software (NIH, 1.52a) and tabulated as a percentage against the control using Microsoft™ Office^© 2020^. 

### 2.13. Radial Migration Assay

C6 tumors were made by the hanging drop method. 10 × 10^4^ cells in 20 μL culture medium were placed as drops on the lower surface of the cap of a 6-well plate, the cap was gently inverted and closed with 3 mL PBS in each well of the plate. These plates were incubated in a CO_2_ incubator for 4 days, after which the tumors were gently reverted into a new plate with culture medium in each well. After 48 h when the tumors adhered to the surface, they were treated with either 2, 8 or 32 μM resveratrol-supplemented medium for 72 h. Cell pictures were taken on the fifth day under a phase-contrast microscope. Length to which the cells in the tumor radially migrated was calculated using ImageJ software (NIH, 1.52a) and tabulated as a percentage against the control using Microsoft™ Office^© 2020^. The radius of migration in control was taken as 100%.

### 2.14. Cell Cycle Analysis

C6 cells were synchronized by serum starvation (cells were cultured in 10% FBS for 24 h, followed by 5% FBS for 24 h, 1% FBS for 24 h, 0% FBS for 24 h, and finally 10% FBS for 24 h). Cells (5 × 10^5^/well) were then plated in 6-well plates and allowed to settle overnight followed by treatment with various doses of resveratrol for another 48 h. For cell cycle analysis, control or treated cells (2 × 10^5^/mL) were re-suspended in 300 μL PBS, fixed by slowly adding 700 μL ice-cold 100% ethanol, and were then stored at −20 °C overnight. The next day, cells were centrifuged at 500 rpm and washed with PBS. RNAse (Thermo Fisher Scientific, EN0531) was added to a final concentration of 100 μg/mL and incubated at 37 °C for 90 min. Suspensions were then centrifuged at 500 rpm, 4 °C for 10 mins. Cells were re-suspended in 200 μL of Cell Cycle Reagent (Guava, 4500-0220) and incubated at room temperature in dark for 30 min, diluted in 1 mL PBS and subjected to cell cycle analysis using Guava™ PCA Systems following manufacturer’s instructions. Data were analyzed and tabulated as a percentage of cells in various cell cycle phases using Microsoft™ Office^© 2020^. Total number of cells in each group were considered as 100%

### 2.15. Annexin V Assay

Cells (2 × 10^5^/well) were plated in 6-well plates and allowed to settle overnight, followed by culture of cells in medium with/without 2 μM resveratrol (pretreatment) for 24 h. Cells and its culture medium were then taken up for annexin V assay using Guava^®^ Nexin Reagent (Luminex Corp., 4500-0450) following the manufacturer’s protocol and the early apoptosis was calculated in percentage and plotted against the control using Microsoft™ Office^© 2020^. Histogram was plotted together for each group taking total live cells as 100%.

### 2.16. Statistical Analysis

All the experiments were performed in at least 3 independent biological replicates. All quantifications were performed using ImageJ software (NIH, 1.52a); all calculations were done using Microsoft™ Office^© 2020^. Statistical significance was calculated by an unpaired t-test of GraphPad^^®^^ software (2018) using mean, SD, and N from three independent experiments, and shown as *p < 0.05, **p < 0.01, ***p < 0.001, *ns = not significant*.

## 3. Results

### 3.1. Cytotoxic and Non-Cytotoxic Doses of Resveratrol as Determined by Cell Viability Assays

We first performed C6 cell viability assay to determine the cytotoxic doses of resveratrol. As shown in Figure S1A, resveratrol showed dose-dependent cytotoxicity in short term (48 h) treatment. TIG3 (normal human fibroblasts) cells was used to compare and obtain relatively safer dose for normal cells. Of note, at doses such as 10, 40 and 160 μM, whereas TIG3 cells did not show toxicity, C6 cells showed dose-dependent response and the cell viability were decreased by 40% at 160 μM treatment. We performed long term proliferation assays taking very low (IC_01_) dose (2 μM). C6 cells, when treated with 2 μM resveratrol for 6 days continuously, remained unaffected (Figure S1B). There was no significant change in cell cycle progression of treated versus control cells (Figure S1C). Annexin V staining assay also did not reveal any sign of apoptosis at this dose (Figure S1D). Based on these data, 2 μM dose of resveratrol was taken further for various cell-based assays.

### 3.2. Resveratrol Protected the C6 Rat Glioma Cells Against a Variety of Stresses

**Figure 1:**
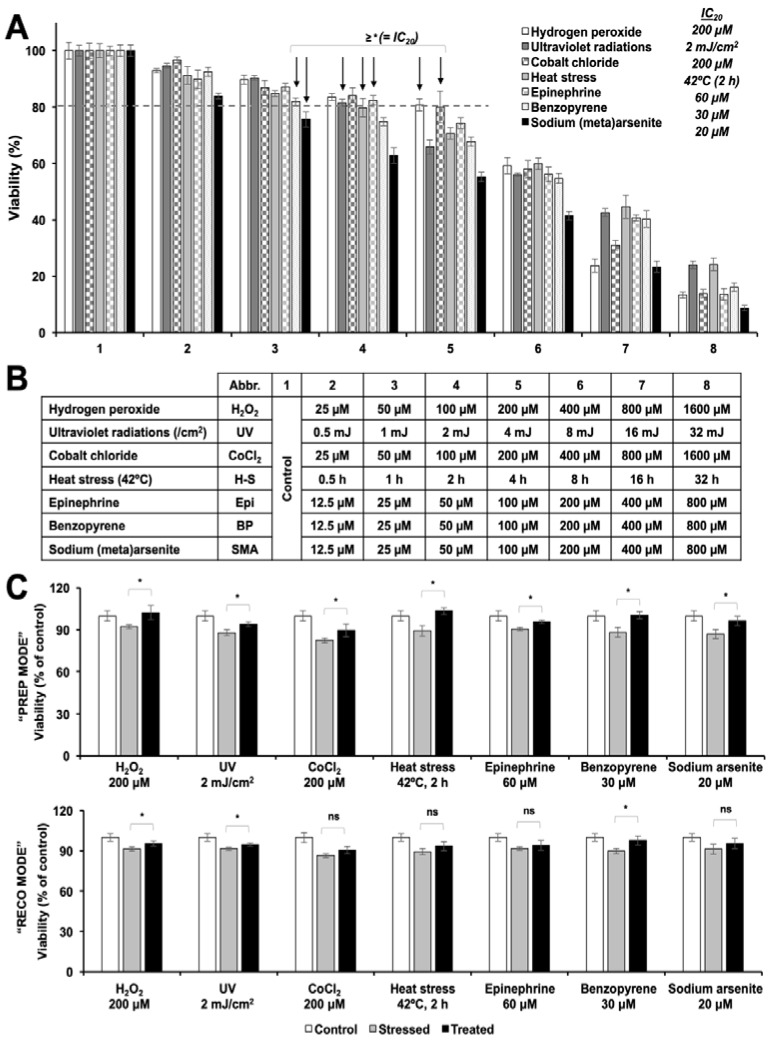
Anti-stress screening of resveratrol in C6 cells. (**A**). Dose-dependent toxicity of chemical stresses listed in (**B**) and identification of their IC_20_ doses using 3-(4,5-dimethylthiazol-2-yl)-2,5-diphenyltetrazolium bromide (MTT)-based cell viability assay. (**C**). Stress protection screening assay showing the anti-stress potential of resveratrol in C6 cells in pretreatment (PREP-defined by 24 h resveratrol pre-treatment followed by stress and recovery in resveratrol supplemented medium) and recovery (RECO-defined by stress followed by recovery in resveratrol supplemented medium) modes. Small but significant protection against all stresses was observed in PREP mode. RECO mode revealed protection against H2O2, UV and Benzopyrene stresses.

To test the anti-stress properties of resveratrol, we selected seven chemical models of stress and tested their dose-dependent effect on C6 viability. By repeated assays, we selected the doses that induced mild stress as determined by only 10–20% decrease in cell viability. Such stress conditions included 200 μM hydrogen peroxide (oxidative stress) for 2 h, 2 mJ/cm^2^ UV-C radiations (DNA damaging solar radiations), 200 μM cobalt chloride (hypoxia) for 2 h, incubation at 42 °C for at 2 h (heat-stress), 60 μM epinephrine (anger and anxiety stress) for 2 h, 30 μM benzopyrene (cigarette smoke stress) for 2 h, and 20 μM sodium (meta)arsenite (heavy metal stress) for 2 h (Figure 1A,B). We used the above-established conditions for challenging the cells and tested the anti-stress potential of resveratrol in two models: (i) PREP mode (defined by 24 h resveratrol pre-treatment followed by stress and recovery in resveratrol supplemented medium), and (ii) RECO mode (defined by stress followed by recovery in resveratrol supplemented medium). In the PREP mode, we found that the resveratrol protected the C6 cells against all the above-mentioned stresses (at IC_20_ doses) to a small but significant amount (Figure 1C). Amongst these, resveratrol showed complete recovery against hydrogen peroxide (~11%), heat stress (~17%) and benzopyrene (~14%). In the RECO mode, resveratrol could significantly protect the cells only against H_2_ O_2_, UV, and benzopyrene stresses. 

### 3.3. Resveratrol-treatment Protected the Cells against DNA Damage, Oxidative, Hypoxia and Protein Aggregation Stresses 

**Figure 2:**
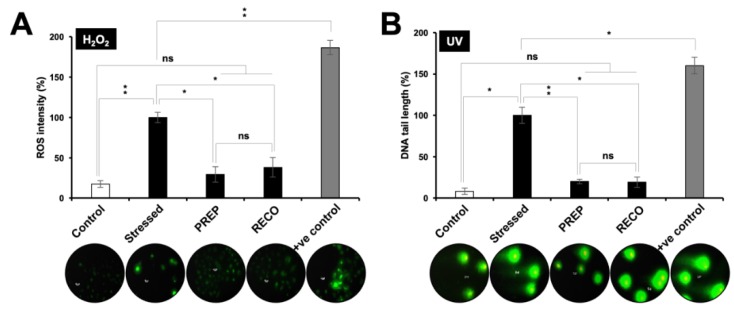
Anti-oxidative stress and DNA damage stress-protective potential of resveratrol. (**A**). Quantitative measure of H_2_O_2_ induced reactive oxygen species (ROS) formation and its suppression in resveratrol treated cells is shown. (**B**). Quantification of UV-C radiations induced DNA strand breaks as determined by Comet assay in control and resveratrol treated cells is shown. A total of 500 μM H_2_O_2_ and 10 mJ/cm^2^ UV were taken as positive controls, respectively. Resveratrol protected the cells against H_2_O_2_ and UV stress both in the PREP and RECO modes of treatment.

Following up on the anti-stress potential of resveratrol for a variety of stresses as described above, we next investigated the mechanism of protection by undertaking the specific biochemical and imaging assays. As shown in Figure 2A, cells treated with hydrogen peroxide (H_2_O_2_) showed induction of reactive oxygen species (ROS). Of note, cells treated with resveratrol either in the PREP or RECO mode showed significant suppression of ROS. Similarly, UV-induced DNA damage was detected by comet assay. Interestingly, resveratrol-treated cells (both in the PREP and RECO mode) showed shorter comet tail suggesting protection against the UV induced DNA damage (Figure 2B).

**Figure 3:**
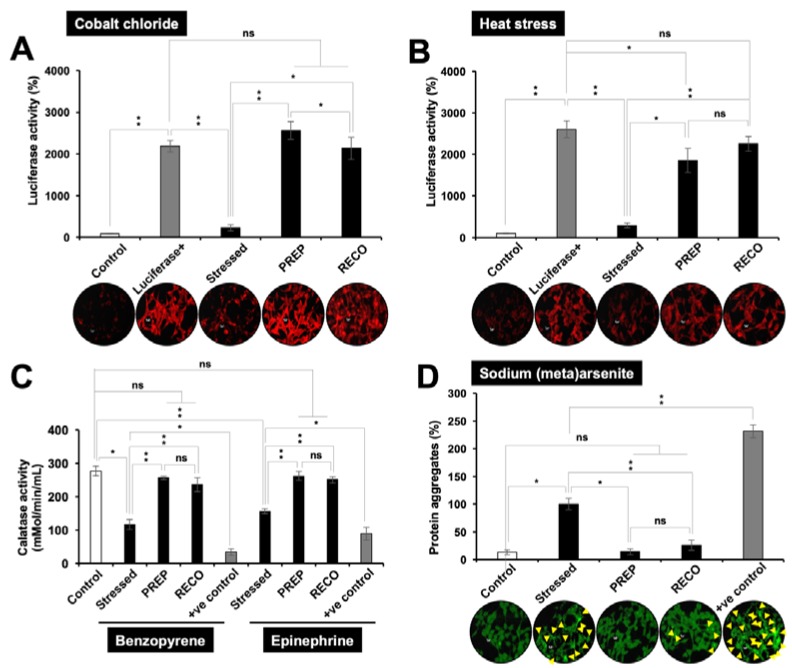
Luciferase reporter assay on control, cobalt chloride (**A**) and heat-stressed (**B**) treated cells in PREP and RECO modes. Relative units of luminescence (luciferase activity (%)) from three independent experiments were plotted. Cells exposed to metal (200 µM cobalt chloride) and heat (42 °C) stress showed inactivation of luciferase activity that was revived in resveratrol-treated cells. (**C**). Benzopyrene (30 µM) and epinephrine (60 µM) suppressed catalase (innate antioxidant enzyme) that was restored in resveratrol-treated cells. (**D**). Sodium (meta) arsenite (20 µM) caused aggregation of GFP (shown by yellow arrowheads) that was de-aggregated in resveratrol-treated cells. A total of 250 µM benzopyrene and 300 µM epinephrine, and 150 µM sodium (meta)arsenite were taken as positive controls in (**C**) and (**D**), respectively.

For assessment of protection against hypoxia and heat stress-induced protein misfolding/aggregation, specific and sensitive reporter assays were performed. Cells were transfected with pGL4-Luc reporter plasmid expressing luciferase protein and then subjected to either of these stresses in the PREP and RECO mode. The expression of luciferase protein was quantitatively detected by reporter assay. As shown in Figure 3A,B, Luciferase activity showed a decrease in cells stressed with metal (cobalt chloride) or heat. A significant increase was observed either in PREP or RECO mode for both cobalt chloride and heat challenged cells. For the cigarette smoke and anger, cells were subjected to benzopyrene and epinephrine, respectively. Significant suppression in the catalase activity (established marker of antioxidant activity) was observed in stressed C6 cells (Figure 3C). Cells cultured in resveratrol-supplemented medium showed elevated catalase activity. For heavy metal stress, GFP-tagged C6 cells were recruited that allowed direct observation of protein aggregates. As shown in Figure 3D, cells treated with sodium(meta)arsenite showed aggregation of GFP. De-aggregation was observed both in the PREP and RECO modes of resveratrol treatment. Of note, in all the stress models, cells showed better recovery in the PREP than in the RECO mode.

### 3.4. Resveratrol Induced Differentiation in C6 Cells

**Figure 4:**
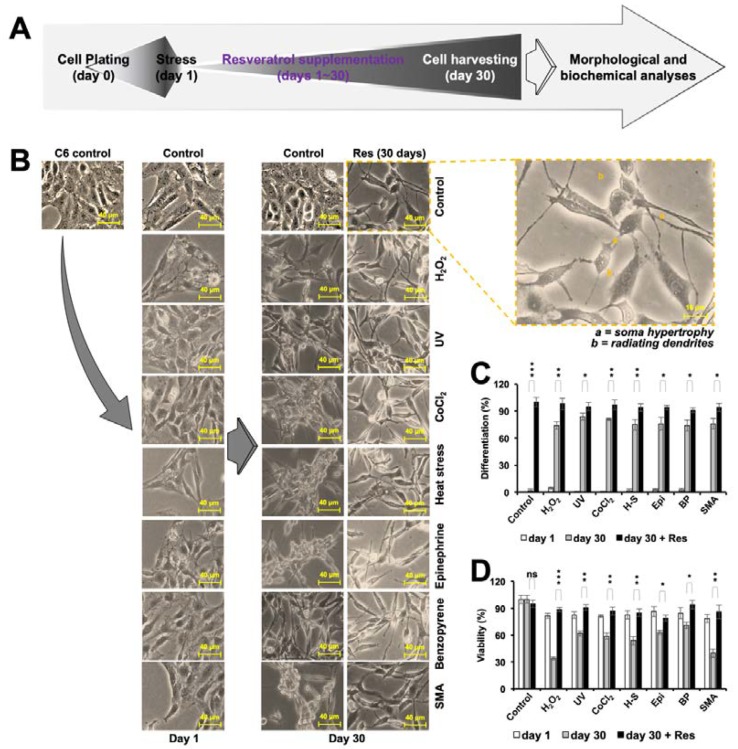
Cell differentiation in glioma upon chronic resveratrol treatment. Flowchart (**A**) and images (**B**) summarizing morphological features of C6 cells, before and after induction of stress, throughout 30 days treatment with resveratrol (Res) with/without the prior stressed state. (**C**). Histogram showing the extent of differentiation (percent differentiation) as evaluated from three independent experiments. The number of cells showing differentiated morphology out of 100 cells from three random fields was plotted on a histogram, where control was taken as 100%. (**D**). Histogram showing loss of viability of C6 cells in response to stress followed by recovery with resveratrol treatment.

Based on the above results and the fact that these stresses are known factors for old age-related brain pathologies (such as Alzheimer’s disease and Parkinson’s syndrome) that are often accompanied by decreased differentiation and functional capacity, we next investigated the effect of resveratrol for C6 rat glioma differentiation. The scheme demonstrated in Figure 4A was followed for treatment. Cells were cultured in resveratrol (2 μM)-supplemented medium for 30 days. As shown in Figure 4 (top-right corner), cells exhibited features morphologically similar to the functional glia (soma hypertrophy and radiating dendrites) by the end of 30 days of treatment (Figure 4B,C). To investigate if the resveratrol could trigger the differentiation signaling in stressed cells, we subjected the C6 cells stressed with various kinds of stresses defined earlier to the non-toxic dose (2 μM) of resveratrol. Morphologically, as shown in Figure 4B, stressed cells also showed differentiated attributes (Figure 4B,C), along with significantly improved cell viability (Figure 4D). 

**Figure 5:**
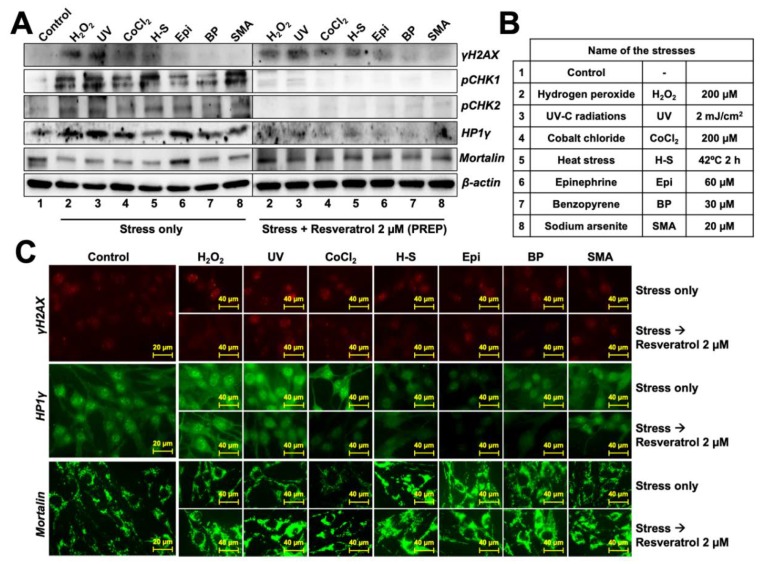
Molecular changes in control and resveratrol-treated stressed cells. (**A**). Western blot showing induction of DNA damage signaling and growth arrest with various chemical stresses listed in (**B**). Resveratrol-treated cells showed considerable reversal of the molecular changes. (**C**). Immunostaining showing induction of DNA damage (γH2AX) and senescence (HP1γ) in stressed cells. Resveratrol-treated cells showed considerable recovery. The decrease in mortalin in cells stressed with some stresses was recovered with resveratrol treatment.

Since the phenotypic recovery was more prominent in the pre-treated cells (Figure 1C), we chose to study the underlying anti-stress mechanism of resveratrol with the “PREP” mode. At the molecular level, the stressed cells showed an increase in γH2 AX (DNA damage marker) particularly in H_2_O_2_ and UV treated cells, both in Western blot and immunostaining assays (Figure 5 and Figure S2). Of note, the PREP cells showed a considerably significant decrease in these markers suggesting protection against stress at the molecular level. Furthermore, whereas cells challenged with each of the stress showed an increase in pCHK1/2 and HP1γ proteins, PREP cells did not show such increase in these proteins endorsing the inhibition of stress-induced DNA damage and premature senescence signaling, respectively. Stress chaperone mortalin showed downregulation in cells treated with all, but epinephrine, stresses. Interestingly PREP cells showed recovery and induction of mortalin in all the stress types. 

### 3.5. Resveratrol-Induced-Differentiation in C6 Cells Was Time-Dependent and Biphasic 

**Figure 6:**
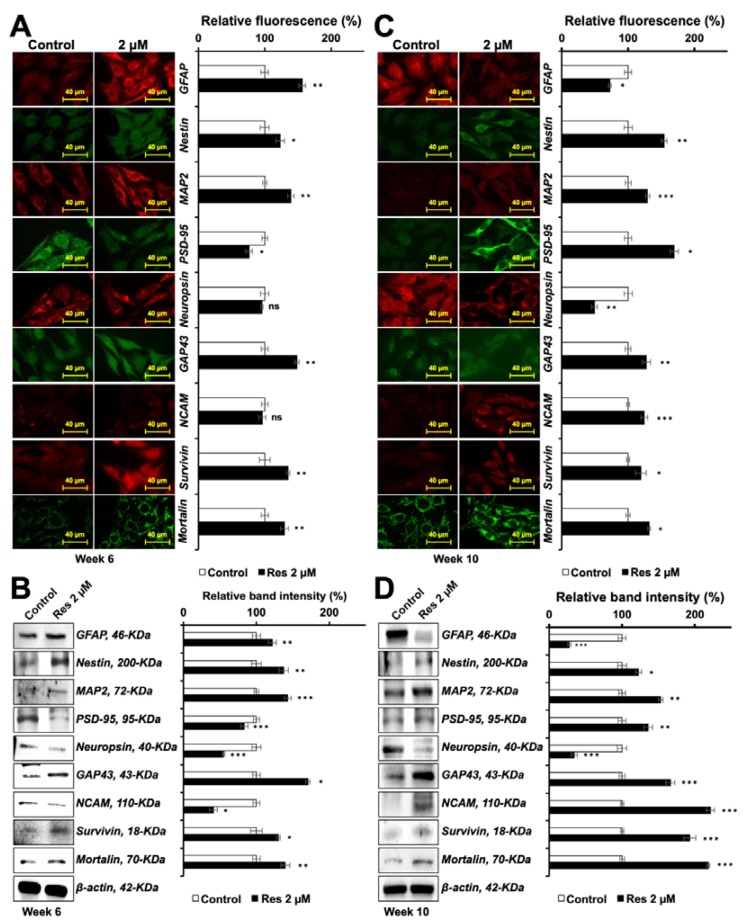
Molecular basis for biphasic differentiation induced by resveratrol (Res) in C6 glioma: Immunostaining and Western blotting (quantification shown in histograms) for proteins specifically expressed in glial and neuronal cells. C6 cells treated with resveratrol for 6 weeks (Glial phenotype, **A** and **B**) and 10 weeks (Neuron phenotype, **C** and **D**) were used.

**Figure 7:**
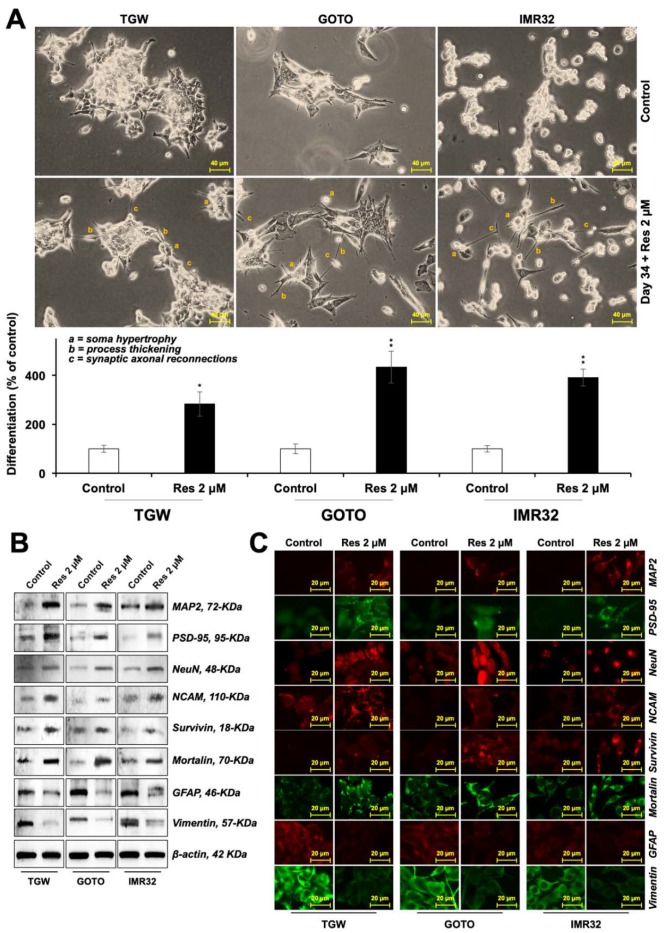
Resveratrol caused differentiation in human neuroblastoma cells. (**A**). Images summarizing cell morphology in the control and resveratrol (res)-treated cells for 34 days. The number of cells showing differentiated morphology out of 100 cells from three random fields were plotted on a histogram, where control was taken as 100%. Western blotting (**B**) and immunostaining (**C**) results showing induction of neuronal-type differentiation in resveratrol-treated human neuroblastoma cells along with the suppression of glial-related proteins.

As shown above, resveratrol-treated C6 cells showed astrocytic morphology at four weeks. On continued culture in the resveratrol-supplemented medium for another 3–5 weeks, we observed neuron-type morphology (soma hypertrophy, process thickening, and synaptic axonal reconnections) of the cells. Based on these observations, we harvested cells for protein analysis at early (6 weeks) and late (10 weeks) periods from the day of the first treatment. As shown in Figure 6A,B, early harvested cells showed a molecular pattern corresponding to immature astrocytic or mixed differentiation (elevated GFAP, Nestin, MAP2, GAP43, Survivin, and Mortalin). PSD-95 and Neuropsin and NCAM did not show increase; they were rather decreased to a small extent. In the late harvested cells (Figure 6C,D), markers specific for neuronal differentiation including elevated levels of MAP2, PSD-95, GAP43, NCAM, survivin, mortalin and nestin, and downregulation of GFAP and neuropsin were observed. These results collectively suggested that resveratrol not only protected the cells against a variety of stresses but also could trigger time-dependent astrocytic and neuronal differentiation in C6 cells. We also examined the differentiation-inducing potency of resveratrol in human-derived neuroblastoma cells. TGW, GOTO, and IMR32 cells were subjected to resveratrol supplementation for 34 days, by the end of which there were significantly a greater number of cells with morphology corresponding to the mature neurons in the resveratrol-treated compared to the control (Figure 7A). Western blotting and immunostaining of the control and treated cells showed that the neuronal differentiation-specific proteins MAP2, PSD-95, NeuN and NCAM were upregulated. Whereas the expression of survivin and mortalin were seen to be upregulated in resveratrol-treated cells (Figure 7 and Figure S3), there was a clear downregulation of GFAP and vimentin.

### 3.6. Resveratrol Caused Modulation of C6 Cell Migration

**Figure 8:**
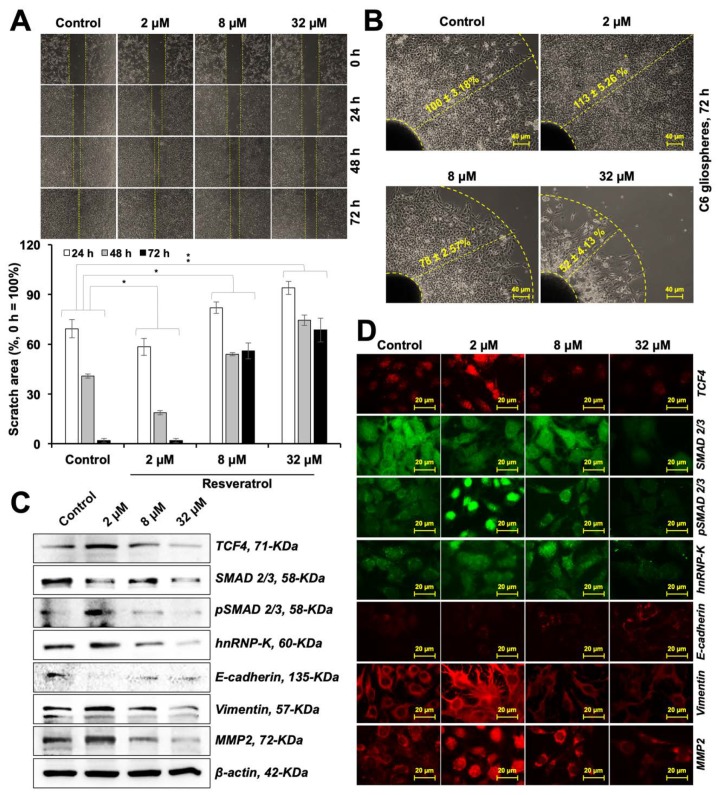
Cell migration analyses of C6 cells upon treatment with resveratrol. Wound scratch migration assay with quantification (**A**) and radial migration assay using tumor spheroids (**B**) showing promigratory response in glioma cells with low dose resveratrol treatment. Western blotting (**C**) and immunostaining (**D**) showing upregulation of canonical markers for migration in cells treated with low dose of resveratrol; the high doses showed downregulation.

Migration and differentiation are vital mechanisms in the embryonic cell development that together determine the fate of cells and thereby develop them into organs. At the time of injury in the adult tissue, migration and simultaneous differentiation of cells constitute the process of regeneration. With these clues, we examined the potential of resveratrol to alter the migration capacity of cells. As shown in Figure 8A,B, the low dose (2 μM) of resveratrol was found to be significantly pro-migratory compared to its higher, but non-toxic, doses by both linear and radial migration assays, respectively. To support this data, we investigated the molecular changes in proteins involved in cell migration and found upregulation of several proteins involved in migration signaling. TCF4, pSMAD2/3, hnRNP-K, Vimentin, and MMP2 showed an increase at the promigratory doses (Figure 8C,D, and Figure S4). Concurrently, E-cadherin was found to be downregulated. Of note, a distinct decrease in upregulated (promigratory) proteins was observed at higher (anti-migratory) doses. Taken together, these results suggested that the differentiation-inducing dose of resveratrol caused upregulation of migratory signaling in glioma cells and converse at the higher doses. 

## 4. Discussion

Stress, largely defined by a feeling of emotional or physical tension, is evoked by any change in the environment that requires adjustment or response. Just like organisms, the cells in culture respond to and offer an established model to stress biology and its intervention. The prime organ that is involved in sensing and responding to stress is the brain. It has been established that the brain functions deteriorate with prolonged exposure to a variety of stresses [12]. Brain-derived cells in culture have been established as a reliable model to study the molecular mechanism of stress-induced damage and its prevention/recovery by natural and synthetic reagents [5,15,39]. In these premises, we used C6 cells in conjunction with a variety of stress models including oxidative stress, smoke, hypoxia, heat-stress, metal and emotional stress to investigate the anti-stress potential of resveratrol. We used hydrogen peroxide supplementation and exposure to UV radiations for oxidative stress. For cigarette smoke, benzopyrene is implicated as a strong carcinogenic model [3]. Its metabolite, benzopyrene-7,8-diol-9,10-epoxide, is known to react to the DNA guanidine to form deoxyguanosine-DNA adducts. Cobalt chloride, used as the model for hypoxic stress, has been shown to cause protein aggregation and misfolding, activate hypoxia-inducible factors, and several proto-oncogenes leading to aggressive proliferation and metastasis of cancer cells [4]. Heat stress has been shown to cause misfolding of proteins leading to the abnormalities in the cytoskeleton and cell functions. Heavy metal poisoning has been shown to cause aggregation and sedimentation of proteins called *p*-bodies or stress granules [5]. These granules are known to cause permanent proteinopathies, not limiting to amyloidosis and dementia. Adrenaline or epinephrine is one of the shock-response α-/β-adrenergic agonist catecholamines secreted by the adrenal medulla in times commonly referred to as three Fs (flight, fight, and fright) [6]. It stimulates the surface α- and β-adrenergic receptors and results in vasoconstriction, bronchodilation, and intra-ocular hypotension.

In our analyses, we found that the level of ROS that mediates a variety of stress responses, decreased in PREP cells and the antioxidant defense signaling showed upregulation suggesting that the resveratrol has the potential to protect cells against the insult from stress and extend their life-span. Of note, resveratrol-treated cells showed a decrease in DNA damage as judged by the level of marker proteins. Collectively, these data strongly suggested the anti-stress potential of resveratrol that may connect to its anti-ageing potential. Indeed, the HP1γ (a marker for cellular senescence) was significantly downregulated in stress-challenged and resveratrol-recovered cells. These findings are supported by earlier studies that have reported anti-stress effects of resveratrol in different stress models and assays [36,40,41,42]. Furthermore, we found that the cells treated in PREP mode showed better recovery, suggesting that the resveratrol may be useful for prophylactic therapy and improving the quality of life. Since aggregation of molecular damage has been established as a hallmark of old age-related brain pathologies including Alzheimer’s and Parkinson’s diseases, we examined the effect of resveratrol on protein aggregation and detected that it has potential to cause de-aggregation of the proteins and correct their misfolding 

Functional properties of cells depend on their differentiation. C6 cells are known to differentiate into astrocytes. Astrocytes, or glia, play a central role in (1) providing anchorage and nutrition to neurons and Schwann (characterized by expression of MAP2, NF200, PSD-95, GAP43 and NCAM), ganglionic (characterized by Neuropsin) and other brain cells, (2) sensing glucose levels and its maintenance, (3) structural constitution of the blood-brain barrier, (4) hormonal and ionic regulation for the brain cells, (5) neural repair and myelination, and (6) circadian clock/biorhythm regulation [12]. Clearly, they are essential in their functional form. Gliomas represent de-differentiated forms that lose these functions and proliferate into a mass of cells/tumors; their occurrence compromises the site-specific functions of the brain affected. In the present study, we found that the resveratrol caused early and functional differentiation of C6 cells, as characterized by the expression of several glial differentiation marker proteins Furthermore, we discovered that the resveratrol-treated C6 cells also differentiated into neuron as defined by the expression of marker proteins. As shown in Figure 6, the expression of GFAP was upregulated, whereas that of PSD-95 and NCAM was downregulated during the six weeks of treatment, when the cells showed glial characteristics. In the same cultures with an extended treatment of resveratrol, expression of neuron-specific marker proteins was observed. Glial- and neuronal-differentiation have been suggested to be associated with enhanced migratory potential via the canonical Wnt/β-catenin signaling [43,44]. Indeed, we observed that the low dose of resveratrol that caused anti-stress effects and induced differentiation in C6 cells caused upregulation of proteins involved in cell migration. These included Wnt/β-catenin signaling, hnRNP-K, Vimentin and MMP2. Both linear and radial cell migration assays showed a significant increase in the migration capacity of resveratrol-treated cells (Figure 8A,B and Figure S5). Of note, the resveratrol-induced differentiation was not restricted to C6 cells, an equivalent effect was observed in human neuroblastoma cells Human brain tumors (glioma and neuroblastoma) are often present as very aggressive forms of cancer, claiming for 55.7 lives in every million globally, ranging from 7.1 per million in Southeast Africa to 6.89 per million in Southern Europe [45]. Glioma has been recognized as one of their most prevalent and deadliest forms with extremely poor prognosis and poor survival and a high prevalence of metastasis. These refract to the conventional therapies including chemotherapy, radiotherapy and surgical regimes. Furthermore, since mature neurons do not replicate it is difficult to rejuvenate their void. Differentiation therapy is deemed to be a treatment of choice. Amongst the differentiation-inducing molecules, natural molecules are more favored than the synthetic for newer anticancer applications because they have broad-spectrum, fewer side effects, possess slow yet long-lasting effects, more affordable/accessible, and can also be used for preventive therapy. Several molecules have been suggested for this purpose. We previously showed the potential of natural compounds like the marine carotenoids and cucurbitacin B-withanone cocktail to induce astrocytic morphology and mixed type biochemical differentiation in rat glioma cells [5,15]. Proteins with the most remarkable changes included GFAP (indicating astrocytic differentiation) and MAP2 (indicating neuronal differentiation). In the present study, resveratrol caused molecular changes in rat glioma cells that led to time-dependent astrocytic differentiation and neuronal differentiation. Phenotypic differentiation was endorsed by molecular markers of both glial and neuronal differentiation. Complimentary to our findings, induction of neuronal differentiation in stem cells has also been reported with resveratrol treatment [46,47]. Furthermore, mortalin, a stress chaperone, reported being crucial for stress recovery, proliferation, protection against oxidative stress showed an increase in response to resveratrol treatment. Several studies have reported the downregulation of mortalin in old age-related brain pathologies, such as Parkinson’s and Alzheimer’s diseases [48,49]. In these premises, upregulation of mortalin and survivin in resveratrol-treated cells suggested their anti-stress and therapeutic potential for old age-related brain pathologies.

## 5. Conclusions

As summarized in the graphical abstract, we show that the resveratrol, at its low (non-toxic) dose, protected C6 cells against several types of stresses and acclimatized them to differentiate into first immature astrocytic followed by neuronal phenotypes. These findings were supported with molecular changes that defined the recovery of cells from stress and their differentiation to glia and neurons.

## Supplementary Materials

The following are available online at https://www.mdpi.com/2072-6643/12/3/671/s1, **Figure S1**: Dose-dependent effect of resveratrol on viability of rat glioma (C6) and human lung fibroblasts (TIG3) cells for determination of nontoxic and safe doses. A. Cells treated with indicated doses of resveratrol for 48 h (A) and 6 days (B) were subjected to MTT-based cell viability assay and Crystal violet staining, respectively. Cell cycle assay (C) and Annexin-V apoptosis assay (D) in control and treated C6 cells are shown, **Figure S2**: Quantification of Western blotting (A) and immunostaining (B) assays in Figure 5A,C, respectively, **Figure S3**: Quantification of Western blotting (A) and immunostaining (B) assays in Figure 7B,C, respectively, **Figure S4**: Quantification of Western blotting (A) and immunostaining (B) assays in Figure 8C,D, respectively, **Figure S5**: Diagram showing concept of linear and radial migration assays. Primary tumor site in a metastatic model of cancer exhibits linear migration of cells (from the primary tumor to the blood vessels, at large). The secondary metastasized site exhibits distension from the secondary tumor to the surrounding parenchyma. The combination of two assays is anticipated to reveal better judgement of effect of test factors on the total migration capacity of cells.
