# Supplementary figures and images for: Anti-Stress, Glial- and Neuro-Differentiation Potential of Resveratrol: Characterization by Cellular, Biochemical and Imaging Assays
Source: Nutrients. 2020 Feb 29;12(3):671. doi: 10.3390/nu12030671 (PMC7146125; doi:10.3390/nu12030671)

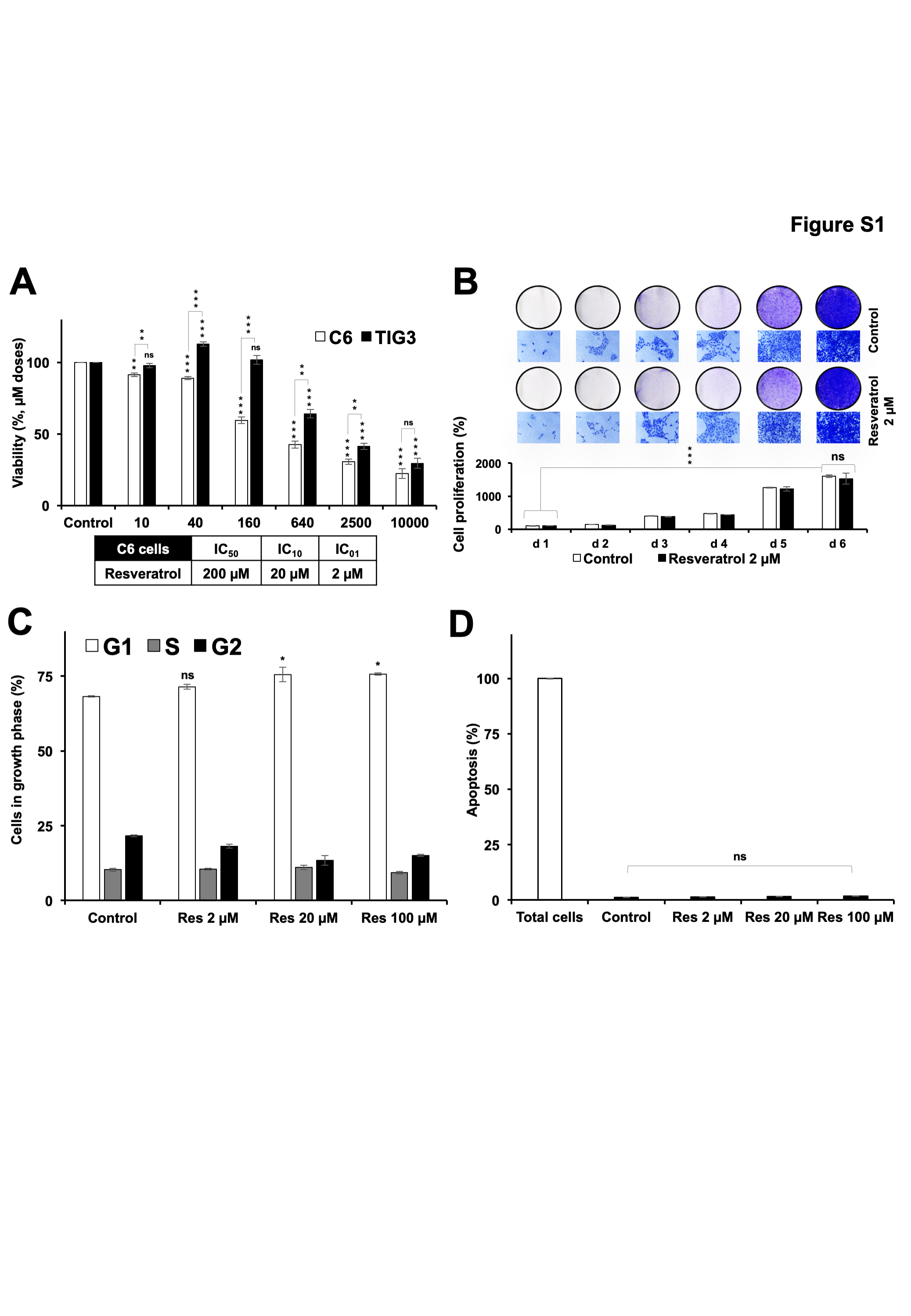

Supplement: Supplementary file 1 [file nutrients-12-00671-s001.zip › Fig. S1.tiff]

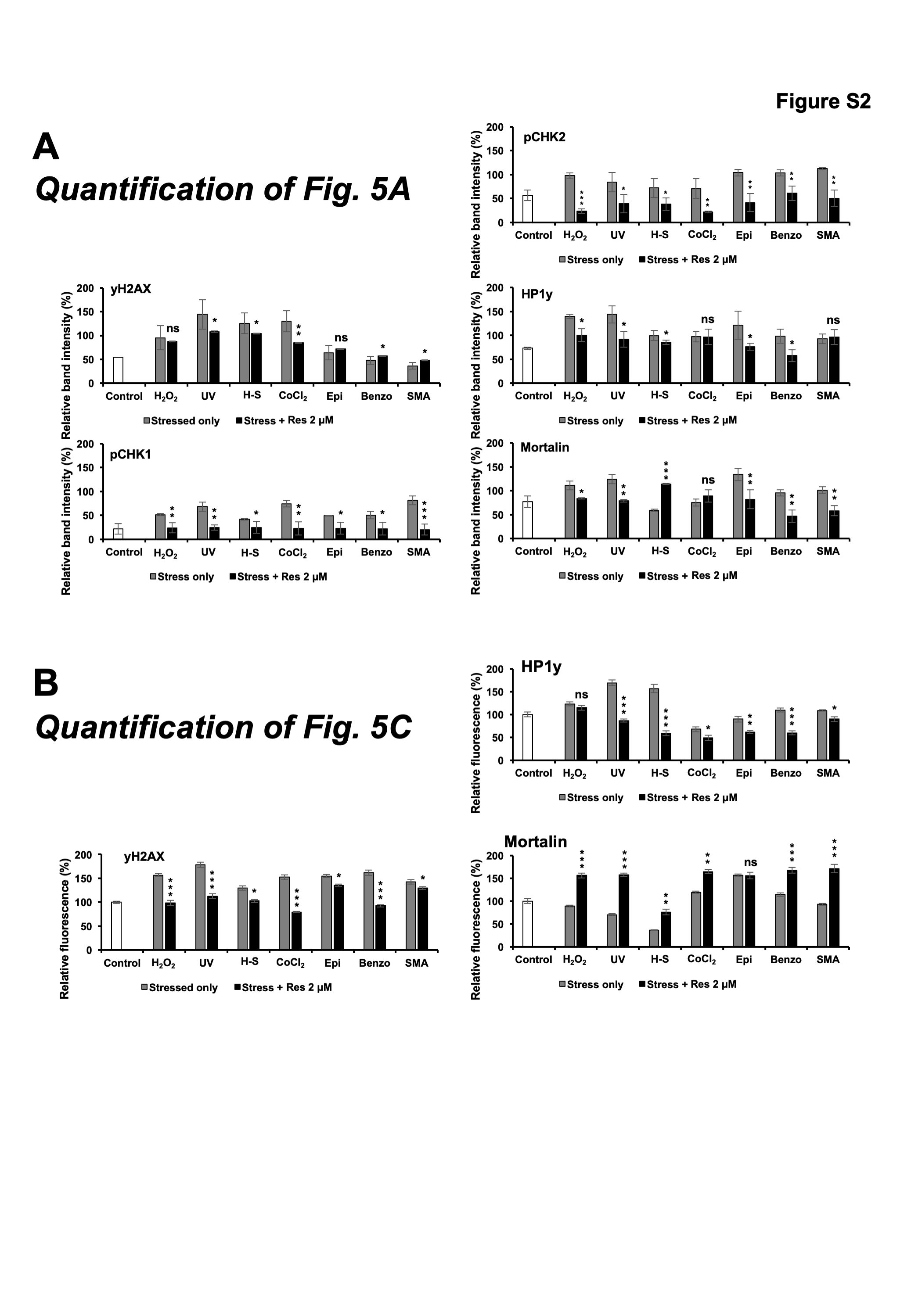

Supplement: Supplementary file 1 [file nutrients-12-00671-s001.zip › Fig. S2.tiff]

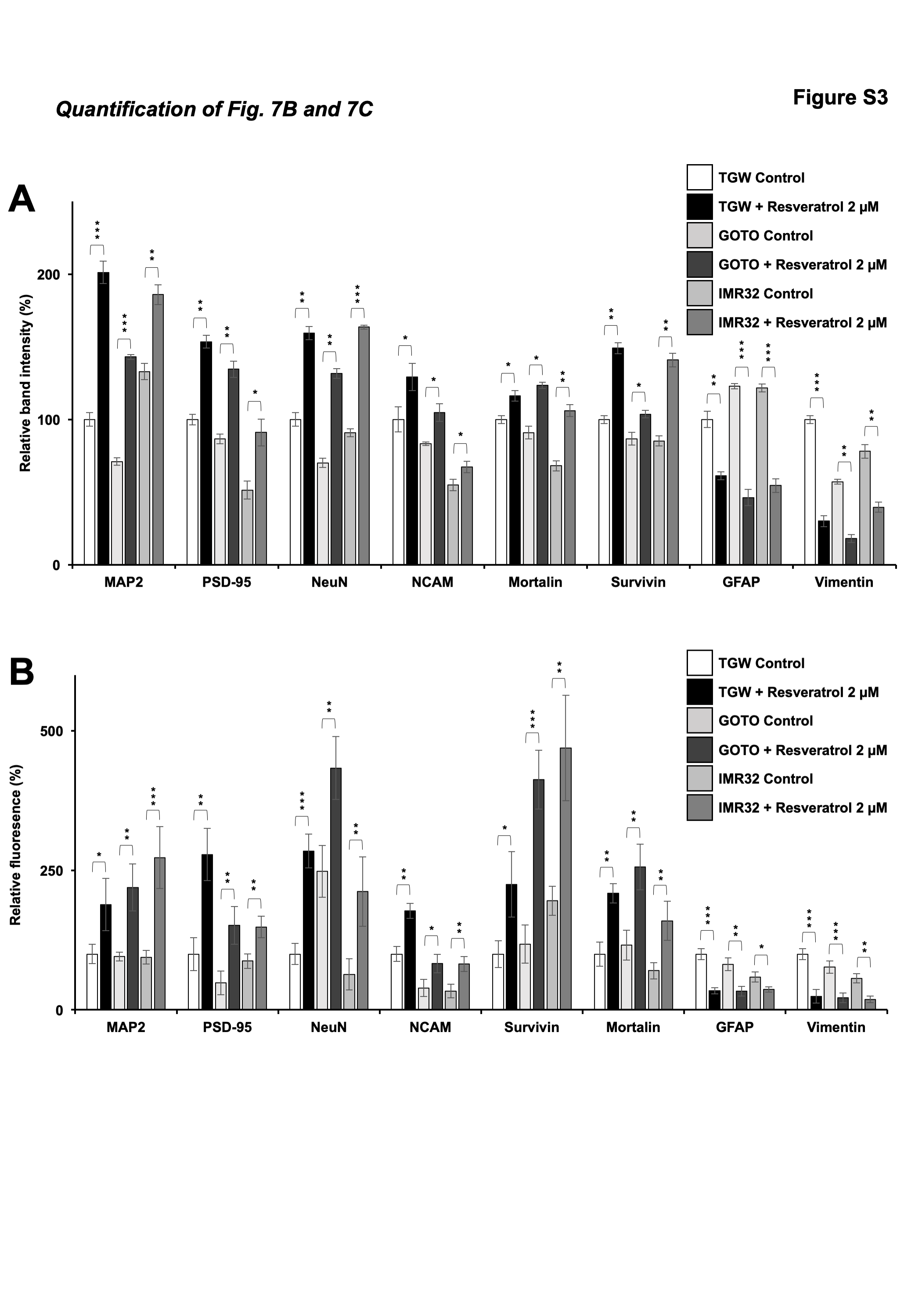

Supplement: Supplementary file 1 [file nutrients-12-00671-s001.zip › Fig. S3.tiff]

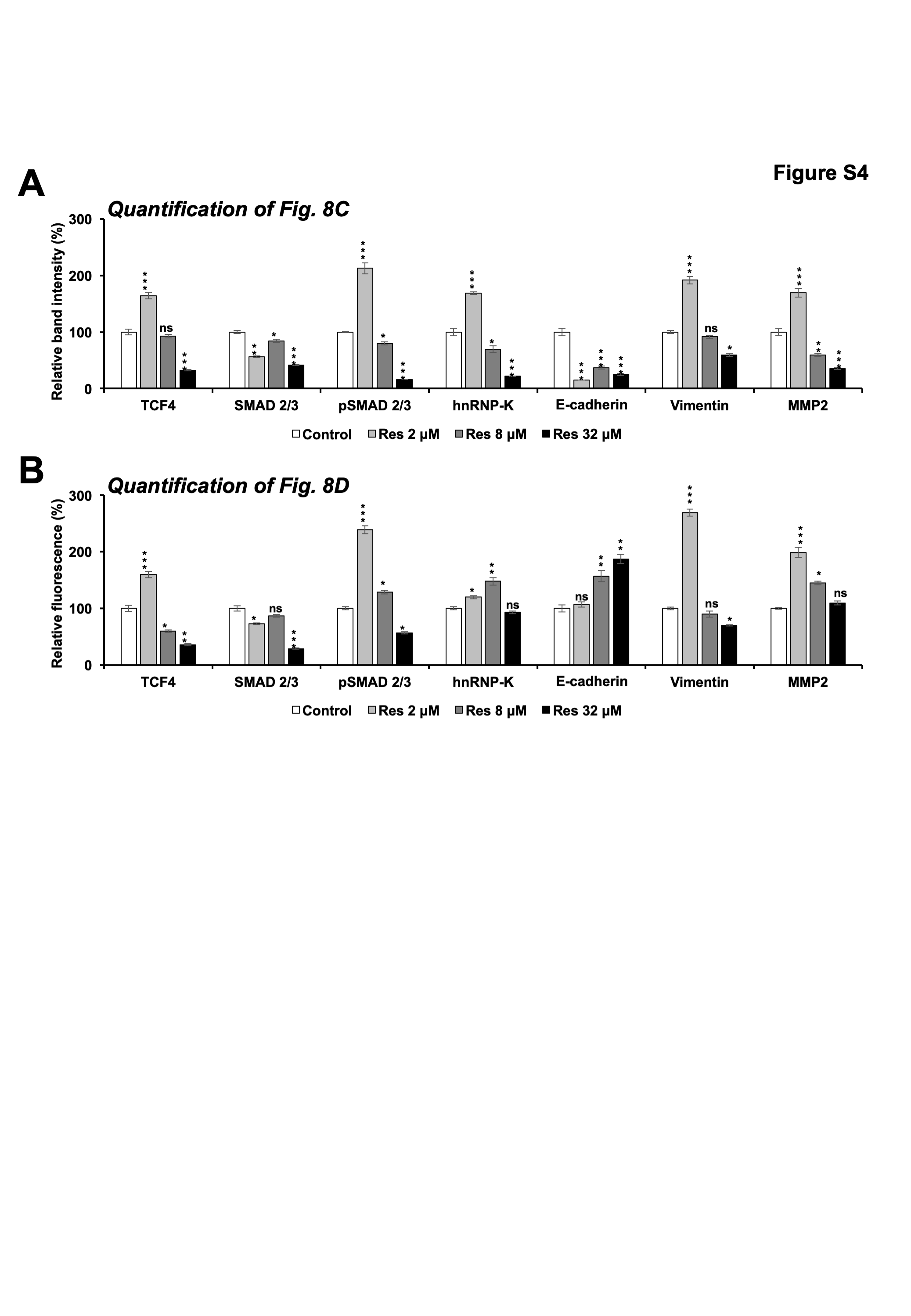

Supplement: Supplementary file 1 [file nutrients-12-00671-s001.zip › Fig. S4.tiff]

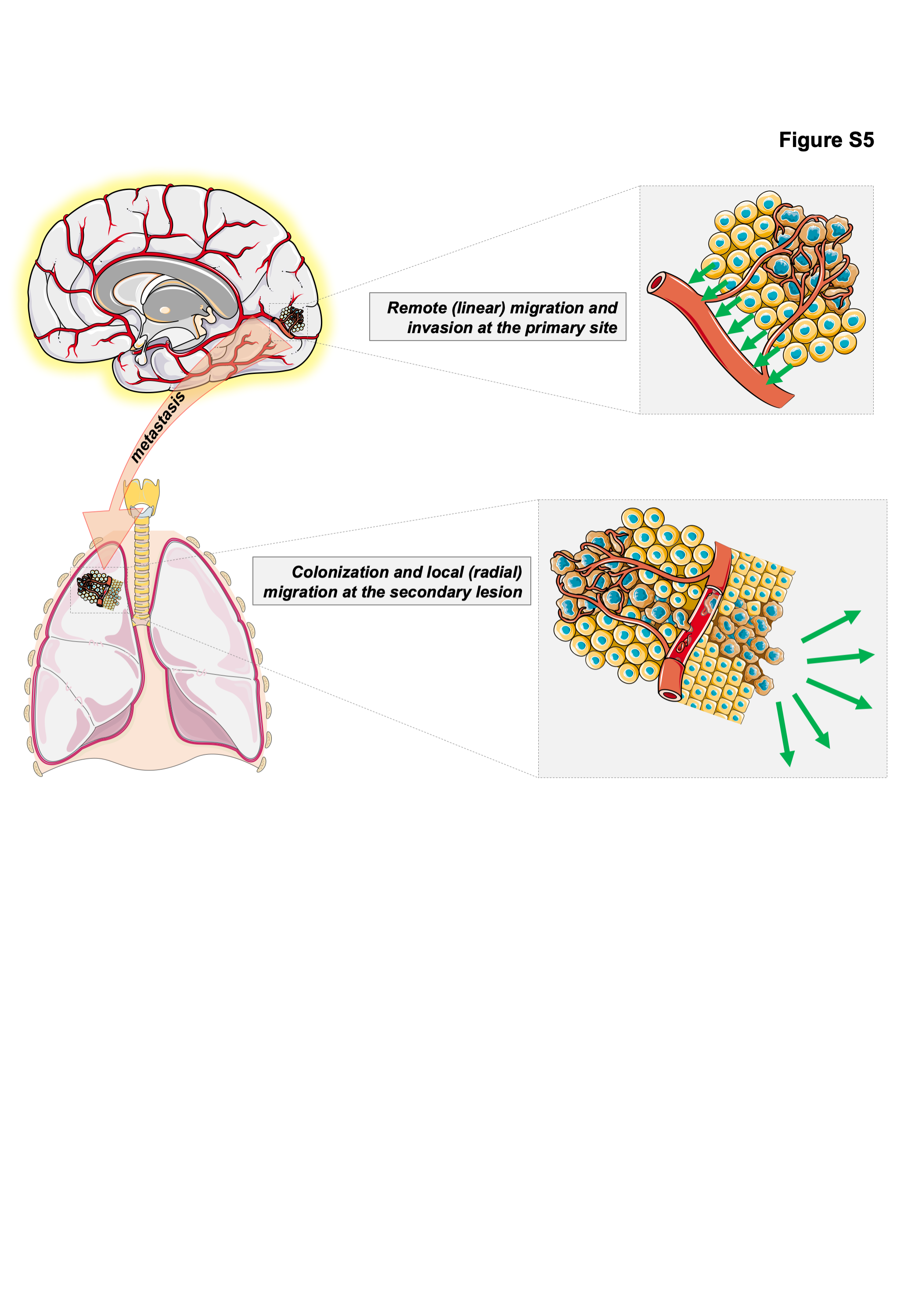

Supplement: Supplementary file 1 [file nutrients-12-00671-s001.zip › Fig. S5.tiff]
